# Identification of *Bna.IAA7.C05* as allelic gene for dwarf mutant generated from tissue culture in oilseed rape

**DOI:** 10.1186/s12870-019-2094-2

**Published:** 2019-11-15

**Authors:** Hongtao Cheng, Fenwei Jin, Qamar U. Zaman, Bingli Ding, Mengyu Hao, Yi Wang, Yi Huang, Rachel Wells, Yun Dong, Qiong Hu

**Affiliations:** 1 Oil Crops Research Institute of Chinese Academy of Agricultural Sciences/Key Laboratory for Biological Sciences and Genetic Improvement of Oil Crops, Ministry of Agriculture and Rural Affairs, Wuhan, 430062 China; 2 Crop Research Institute, Gansu academy of Agricultural Sciences, Lanzhou, 730070 Gansu China; 3 John Innes Centre, Norwich Research Park, Norwich, NR4 7UH UK

**Keywords:** *Brassica napus*, Dwarf, *IAA7*, MutMap, Auxin, Tissue culture

## Abstract

**Background:**

Plant height is one of the most important agronomic traits in many crops due to its influence on lodging resistance and yield performance. Although progress has been made in the use of dwarfing genes in crop improvement, identification of new dwarf germplasm is still of significant interest for breeding varieties with increased yield.

**Results:**

Here we describe a dominant, dwarf mutant G7 of *Brassica napus* with down-curved leaves derived from tissue culture. To explore the genetic variation responsible for the dwarf phenotype, the mutant was crossed to a conventional line to develop a segregating F_2_ population. Bulks were formed from plants with either dwarf or conventional plant height and subjected to high throughput sequencing analysis via mutation mapping (MutMap). The dwarf mutation was mapped to a 0.6 Mb interval of *B. napus* chromosome C05. Candidate gene analysis revealed that one SNP causing an amino acid change in the domain II of *Bna*.*IAA7.C05* may contribute to the dwarf phenotype. This is consistent with the phenotype of a gain-of-function *indole-3-acetic acid* (*iaa*) mutant in *Bna*.*IAA7.C05* reported recently. GO and KEGG analysis of RNA-seq data revealed the down-regulation of auxin related genes, including many other *IAA* and *small up regulated response* (*SAUR*) genes, in the dwarf mutant.

**Conclusion:**

Our studies characterize a new allele of *Bna*.*IAA7.C05* responsible for the dwarf mutant generated from tissue culture. This may provide a valuable genetic resource for breeding for lodging resistance and compact plant stature in *B. napus*.

## Background

Plant height is a key trait related to lodging resistance, harvest index and fertilizer response [1]. Within important crops, such as wheat and rice, the green revolution brought about significant increases in yield by combining the breeding of high yielding dwarf varieties with agricultural mechanisation and fertilizer application [2]. Extreme dwarf varieties are usually correlated with poor agronomic performance and traits such as smaller grains, excessive tillering, or narrow leaves [3, 4], therefore semi-dwarf varieties are used to enhance yield and lodging resistance [5, 6].

Specific genes contributing to plant height are widely used in crop improvement. In rice, the semi-dwarf gene *sd1*, which regulates a key step in gibberellic acid (GA) biosynthesis, has been used worldwide in rice production [5]. The use of *Rht* (Reduced height) genes, involved in GA signaling transduction, was instrumental in bringing about the “green revolution” in wheat as well as other crops [7]. Many phytohormones, including GA, brassinosteriod (BR), strigolactone (SL), auxin, abscisic acid (ABA) and ethelyne (ETH), have been reported to influence crop height [3, 8–12]. Additional dwarfing genes involved in other pathways have also been shown to determine plant height [13]. The mechanisms underlying this complex trait are still largely not understood.

*B. napus* (oilseed rape) is one of the most important oilseed crops in China and the second most important oilseed worldwide [14]. Plant height is a key agronomic trait for rapeseed production as its heavy canopy makes it prone to lodging. Dwarfing can increase both lodging resistance and yield performance [15]. Within *B. napus* several dwarf mutants have been identified and the causal genes have been cloned. Dwarf gene *BREIZH*, derived from oilseed rape through chemical mutagenesis, was mapped by RAPD and RFLP markers [16, 17]. Dwarf gene *Brrga1-d*, encoding a DELLA protein, was first identified in the diploid, *Brassica rapa*, before being transferred to *B. napus* [18, 19]. Genetic analysis showed that mutation of *Brrga1-d* alters GA signaling pathway thus reducing plant height [20]. Semi-dwarf gene *DS-1,* mapped to chromosome A06, also encodes a DELLA protein. The single amino acid substitution of proline to leucine in the VHYNP motif causes a gain-of-function mutation in GA signaling [21]. Another semi-dwarfing gene, *ds-3*, encoding a mutant DELLA protein, also has the substitution of proline to leucine in the conserved VHYNP motif [6]. The recessive dwarfing gene, *BnaC.dwf*, was demonstrated to be insensitive to exogenous GA3 [22]. Dwarf mutant “NDF-1” in *B. napus* was found to be controlled by one major gene with three base pair mutations in the pyrimidine box of *GID1* promoter [23]. Another dwarf mutant with down-curved leaf (*Bndwf/dcl1*) was mapped to a 175 kb region on *B. napus* chromosome C05 [15].

Auxin regulates many aspects of plant development [24]. Auxin signaling controlled by ARFs and Aux/IAA has been well studied in Arabidopsis [25]. Under low auxin concentration, Aux/IAA proteins interact and inhibit the activity of AUXIN RESPONSE FACTOR (ARFs), thereby repressing the auxin response gene expression [26]. At high intracellular auxin concentrations, auxin is perceived by TIR1/AFB1–3 receptors and the Aux/IAA proteins are then degraded by the ubiquitin-proteasome pathway. This releases the repression of ARFs and auxin response genes are activated [25]. Loss-of-function mutants of any of the twelve *Aux/IAA* genes in Arabidopsis do not show an obvious phenotype [26]. However, amino acid mutation in the conserved motif of domain II in Aux/IAA proteins causes dramatic gain-of-function phenotypes [27]. Gain-of-function mutants of 10 out of 29 Arabidopsis *Aux/IAA*, including *iaa1*, *3*, *6*, *7*, *12*, *14*, *17*, *18*, *19* and *28*, have been reported [28–39]. All mutants were caused by one amino acid substitution in the conserved GWPPV motif of domain II. This leads to reduced TIR binding, disrupting the degradation of Aux/IAA and therefore increasing the suppression of ARFs [40, 41].

In the present study, one dominant dwarf mutant was identified during the genetic transformation in *B. napus*. Transgenic element and expression results showed that this effect was not due to T-DNA insertion and therefore was most likely caused by somatic mutation generated during tissue culture. Following the construction of a segregating population, we performed MutMap to map the candidate region to one 0.6 Mb interval on chromosome *Bna*C05. Further, phenotype and correlation analysis of this region was consistent with the region for a *B. napus* dwarfism mutant reported recently [42, 43]. Eighteen candidate genes in this region were found to contain non-synonymous SNPs within coding regions. A single nucleotide substitution (G to A) in the conserved domain II of candidate gene *Bna.IAA7.C05* resulted in changing the GWPPV motif to EWPPV. A C to T substitution in the conserved domain II of *BnaIAA7* changing the GWPPV motif to GWLPV has been shown to cause a dwarfism phenotype recently [42, 43]. Thus, we speculated that another allelic mutation in the conserved domain II of *Bna.IAA7.C05* leads to the dwarfism phenotype of *G7*. Exploitation of this dwarf mutant, with a compact plant stature and reduced plant height phenotype, will be valuable to assist breeding lodging resistant varieties.

## Results

### Generation and phenotype of the G7 dwarf mutant

**Fig. 1:**
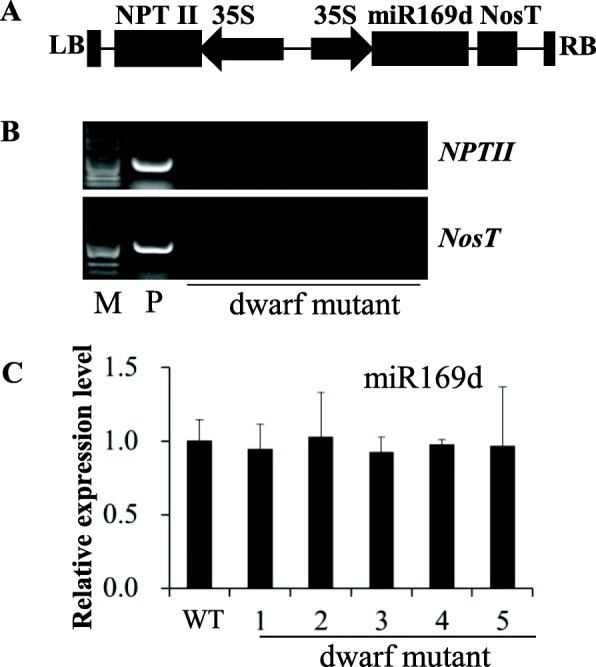
**a**. Schematic representations of the key components of the transformation vector. **b**. Transgenic elements kanamycin (NPTII) and NosT could not be detected in dwarf mutants. P. positive transgenic plant. M. marker. **c**. Expression level detection of miR169d by RT-qPCR showed no difference between G7 dwarf mutants and WT

To determine the function of miR169d in *B. napus*, we previously performed overexpression of miR169d in oilseed rape using the vector illustrated in Fig. 1a. We obtained 14 transgenic plants after genetic transformation, only one of which exhibited a dwarf phenotype with down-curved leaves. Detection for *NPT* and *NOS* presence within the vector (Fig. 1a) showed that the dwarf mutant contained no transgenic element (Fig. 1b), indicating this phenotype was not caused by overexpression of miR169d. Stem-loop RT-qPCR to check expression level of miR169d showed no significant increase in expression (Fig. 1c). Therefore, we hypothesized that this mutant was generated during the tissue culture process.

**Fig. 2:**
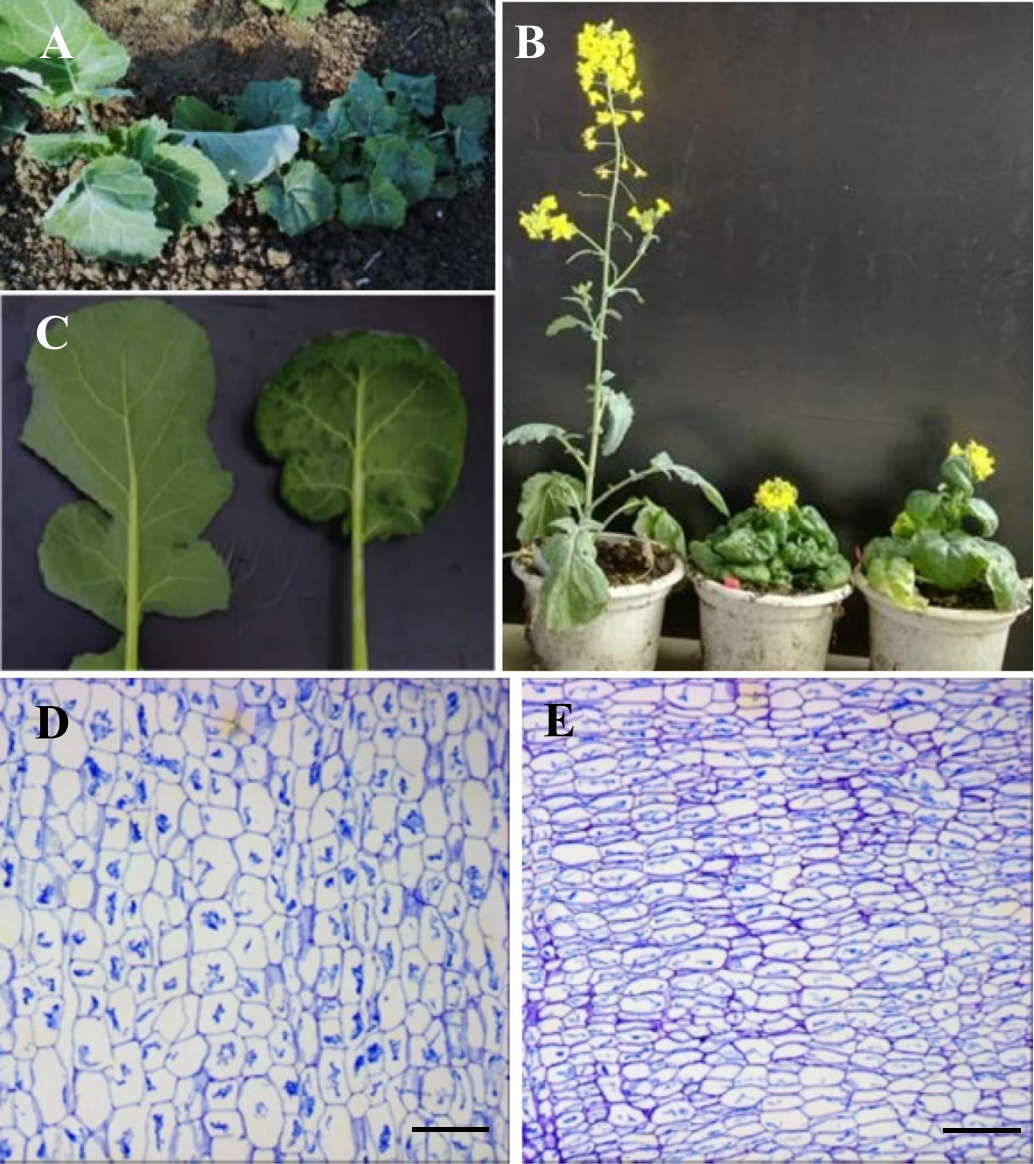
Wild-type and mutant plant phenotype in **a**. the field after one months growth and **b**. the growth chamber. Left = WT, right = G7 dwarf mutant. **c**. The leaves of the G7 dwarf mutant displayed a down curved phenotype. Anatomy of leaves in WT (**d**) and mutant plants (**e**). Bar = 0.1 mm showed significant differences in cell size

This dwarf mutant displayed down-curved leaves and reduced height compared to WT at seedling stage (Fig. 2a). Following the floral transition, mutant plants exhibited a compact and significantly dwarf plant stature (Fig. 2b). Plant height of the dwarf mutant was significantly shorter (~ 30 cm) than WT (~ 150 cm) due to reduction of internode length. The leaves of this dwarf mutant became slightly crinkled and down-curved (Fig. 2c). Microscopy revealed that the cell size in dwarf plant leaves (Fig. 2e) was significantly decreased compared to WT (Fig. 2d).

### Inheritance of the dwarf phenotype

**Fig. 3:**
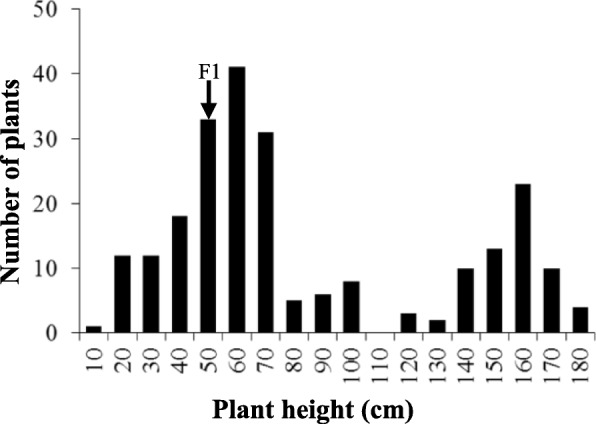
Distribution of plant height in the F2 population derived from the cross of G7 and 48,557 showing bimodal distribution. Arrow indicated the plant height of F1 from dwarf mutant G7 and normal plant 48,557 which was about 50 to 60 cm

The dwarf mutant, henceforth known as “G7”, and the WT variety “48,557” were used to construct a segregating population. The resulting F_1_ plants (48,557 × G7) exhibited down-curved leaves and crinkled phenotype, indicating the G7 mutation was controlled by a dominant gene. Mutant G7 displayed curled and wavy leaves and the yield-related traits were decreased compared with normal variety 48,557. The F1 plants showed intermediate plant height between two parents. Though the branch number and total silique number was decreased, higher density of pod layer were observed in the F1 plants. Plants from the F_2_ population could be divided into two distinct groups: WT (tall plants with normal leaves) and those displaying the G7 phenotype (dwarf plants with down-curved leaves). At seedling stage, 255 plants from the F_2_ population contained 190 G7 plants to 65 WT plants. A Chi-squared test revealed that this segregation pattern agreed with the 3:1 Mendelian segregation ratio (*P* = 0.857 > 0.05, χ^2^_0.05_ = 0.0327 < χ^2^_0.05_ = 3.842). Twenty-four G7 mutant plants from the F_2_ population died before flowering. Plant height of the remaining F_2_ individuals displayed a bimodal distribution (Fig. 3) again demonstrating a 3:1 Mendelian segregation ratio (*P* = 0.2443 > 0.05, χ^2^_0.05_ = 1.356 < χ^2^_0.05_ = 3.842). Therefore, we considered that the G7 dwarfism phenotype is likely controlled by one single dominant gene.

### Identification of candidate genomic region by MutMap

**Fig. 4:**
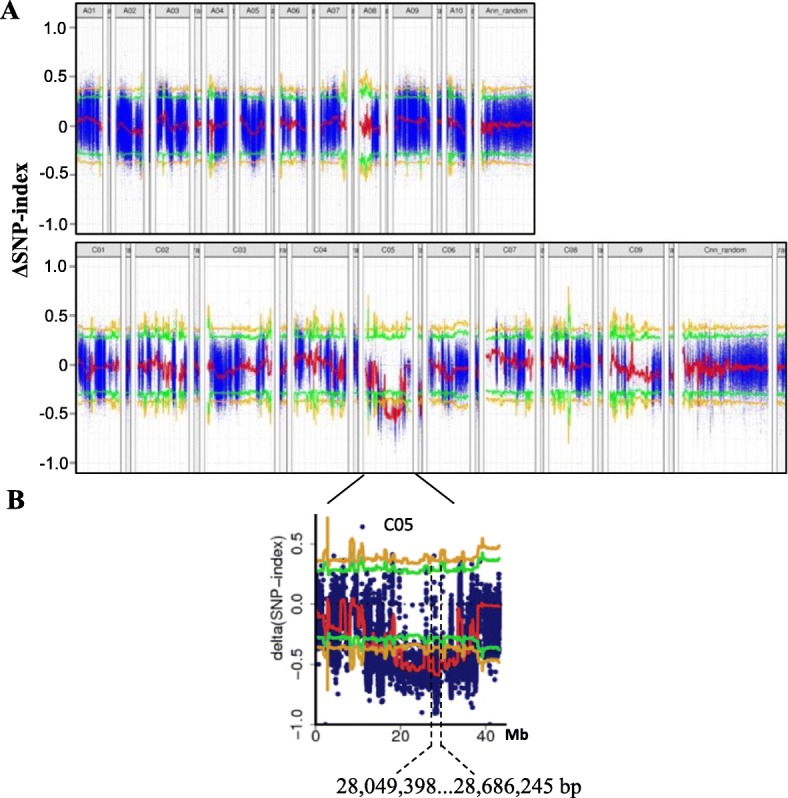
**a**. Plots showing the ΔSNP-index value (blue) and the average value of the ΔSNP-index for all SNP within a 4 Mb sliding window (red) for all chromosome in the *B. napus* A sub-genome (upper panel) and C sub-genome (middle panel). Green and yellow lines indicates the 95% (*P* < 0.05) and 99% confidence level (*P* < 0.01). **b**. The candidate region on chromosome C05 showing a ΔSNP-index peak between 28,049,398 to 28,686,245 bp

Genomic DNA of the two parents (G7 and 48,557) and the two pools (dwarf-pool and WT-pool) was sequenced, resulting in 510,946,104, 523,601,254, 438,302,148 and 411,082,480 clean reads, respectively. After aligning clean reads with the reference genome sequence, we acquired 232,279,673 and 215,670,613 unique mapped reads from dwarf-pool and WT-pool respectively, corresponding to 53 and 52.46% coverage of the genome. Ultimately, 5,388,850 and 5,370,965 SNPs were identified between the two DNA pools and the reference genome. After calculating the SNP-index from the dwarf-pool and WT-pool, the Δ SNP-index was plotted against the A and C sub-genome positions (Fig. 4a). At 99% significance level, one significant locus for dwarf phenotype on chromosome C05 (from 28.0–28.6 Mb) was identified with a peak Δ SNP-index value (Fig. 4b).

### Candidate gene identification by MutMap

**Fig. 5:**
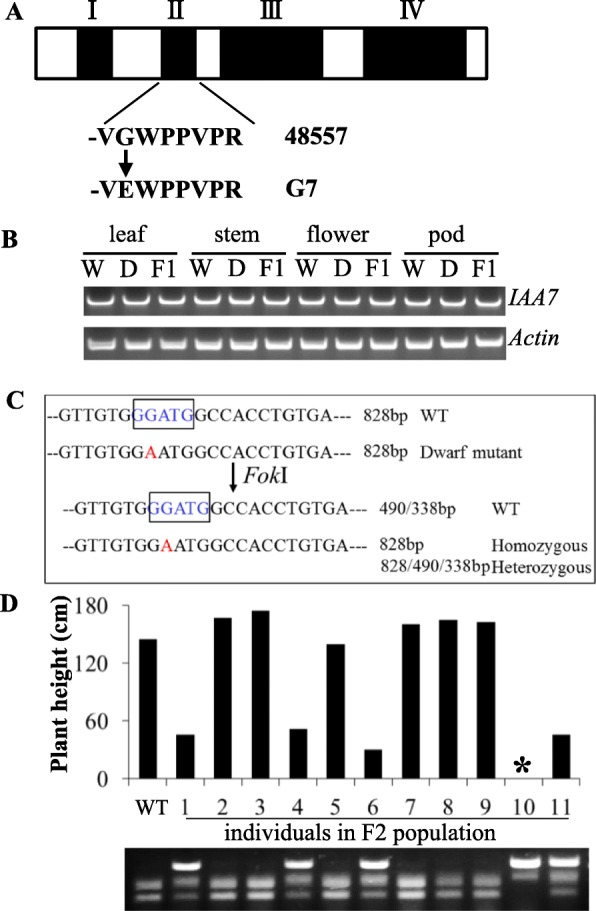
**a**. *IAA7* gene structure is divided into four domains. A SNP identified between the G7 dwarf mutant and WT caused a G (WT) to E (G7 mutant) amino acid substitution within domain II of Bna.IAA7.C05. **b**. Gene expression of Bna.IAA7.C05 between WT (W), dwarf mutant (D) and F1 lines across multiple tissues was detected by RT-PCR. *BnaActin* was used as control. **c**. Variation within the recognition site of restriction enzyme *Fok*I (blue boxed sequence) was used to develop a dCAPs marker for the SNP within *Bna.IAA7.C05*. **d**. Plant height of F2 individuals co-segregated with the dCAPs marker (* plant died at pod maturation)

Within the candidate region on *Bna*C05, SNPs resulting in amino acid variation were identified within 18 candidate genes (Additional file 1: Table S1). Putative gene function prediction revealed none of the candidate genes had been implicated in the control of plant architecture except *BnaC05g29300D*, a homolog of *AtIAA7* in Arabidopsis. The SNP in *BnaC05g29300D* results in a glycine to glutamic acid (G to E) amino acid substitution in *Bna.IAA7.C05* (Fig. 5a) within conserved domain II resulting in the core sequence change from GWPPV to EWPPV. Many auxin gain-of-function mutant alleles of *aux/iaa* which exhibit morphological abnormalities, including decreased apical dominance, reduced plant height and severe stunting, have been reported in Arabidopsis. Thus, this gene is the most likely candidate gene for the G7 dwarf mutation. Expression analysis showed that *Bna*.*IAA7.C05* gene was constitutively expressed in leaf, flower, stem and pod (Fig. 5b). No obvious difference of expression level was detected between the mutant and wild type (Fig. 5b).

### Dwarf phenotype co-segregates with the dCAPs marker for SNP variation in *Bna.IAA7.C05*

The *Bna.IAA7.C05* gene has been cloned from two dwarf EMS mutants and the function confirmed by genetic transformation [42, 43]. Sequence analysis showed that the dwarf phenotype of both these mutants were due to amino acid mutation in the GWPPV motif (GWPPV to GWLPV) of *Bna.IAA7*, thus leading to the same dwarfism phenotype. Based on the SNP within *Bna.IAA7.C05*, we developed a dCAPs marker to classify the individual type from F_2_ population (Fig. 5c). This marker co-segregated with plant height (Fig. 5d) and therefore is a suitable early diagnostic tool for the G7 dwarf mutation.

### RNA-seq analysis revealed enrichment of the auxin signaling pathway in the G7 dwarf mutant

**Fig. 6:**
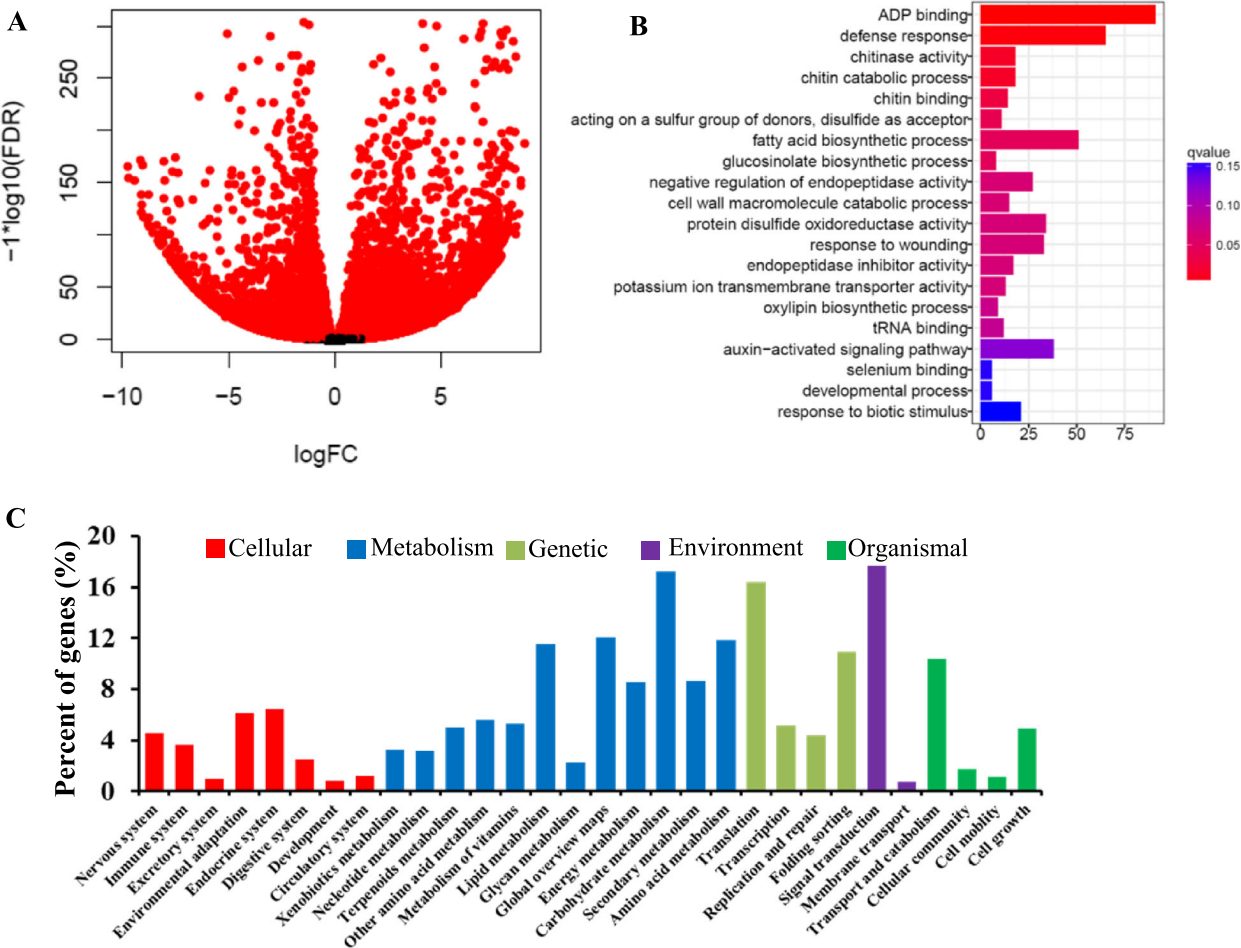
Transcriptome analysis of dwarf mutant and wild type.**a**. Differentially expressed genes detected between G7 and westar. **b**. GO analysis of DEG showing enrichment of the auxin-activated signaling pathway. **c**. KEGG analysis of DEGs showing enrichment of signal transduction within the environment process category

To further understand the regulatory mechanism, we performed transcriptome analysis of the G7 dwarf and WT plants. A total of 9516 differentially expressed genes (DEGs) were found between G7 and 48,557 (Fig. 6a). Within the biological process classification, auxin activated signaling pathway was detected to be overrepresented (Fig. 6b). To investigate the potential role of DEGs, we performed KEGG (Kyoto Encyclopedia of Genes and Genomes) analysis. Five processes including cellular, metabolism, genetic environment and organismal were found to be enriched for DEGs (Fig. 6c). We also found that the signaling transduction pathway category was overrepresented in environment processes (Fig. 6c).

**Fig. 7:**
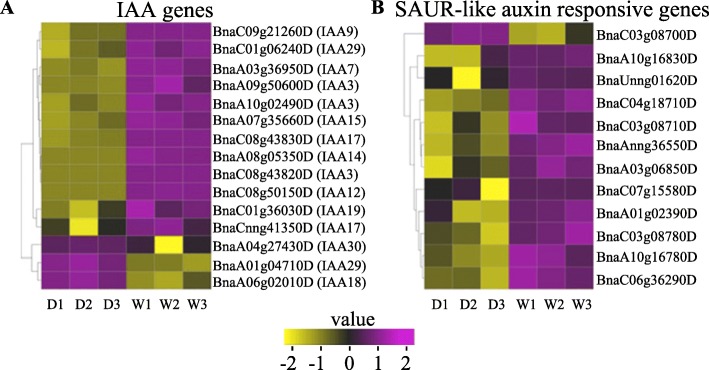
The heatmap of genes that are involved in auxin signaling transduction pathway showing up-regulation (purple) and down regulation (yellow) in dwarf mutants (D1, D2, D3) and WT (W1, W2, W3). Color key represents log2 transformed FPKM (fragments per kilo base of exon per million fragments mapped) values, from low (yellow) to high (purple). SAUR (small auxin up RNAs)

Auxin plays a major role in regulating numerous processes for plant growth and development. Mutation of domain II of IAA proteins significantly suppressed auxin induced expression of other IAA genes in Arabidopsis [41]. In the present study, we found that 12 IAA genes were downregulated in the G7 dwarf mutant compared with WT (Fig. 7a). The expression of small auxin up RNAs (SAUR) was triggered by auxin. Overexpression of various Arabidopsis SAURs leads to induction of cell elongation and growth [44]. The expression level of many *SAURs* genes was also suppressed in the dwarf mutant (Fig. 7b).

## Discussion

Many studies have been performed for plant height, but the application of dwarfing genes is still rare. Newly identified genes for dwarfism offered important sources of variation for breeding crops with lodging resistance and compact plant architecture [45]. Dwarfism and compact mutants have also been shown to possess improved response to fertilizer applications [1]. In the present study, one dwarf mutant was casually obtained in tissue culture when we conducted miR169d overexpression transformation. Genetic variation can be induced by chemical mutagens and physical treatment or tissue culture [46], therefore tissue culture alone or combined with chemical and biological agents can be utilized to increase genetic variability and provide resources for new commercial cultivar production [47, 48]. Within tissue culture itself, mutations can arise from calli, organ cultures, protoplasts and somatic embryogenesis [48]. Mutants induced from somatic variation have been successfully utilized to create potential new varieties in potato, millet and other crops [49–51]. The dwarf mutant generated from tissue culture in our study has the potential for breeding varieties with increased lodging resistance and improved fertilizer response.

MutMap can be used to easily screen and map genes to precise location by sequencing of DNA pools and has been successfully used in many species, such as Arabidopsis, rice, cucumber and oilseed rape [45, 52, 53]. In the present study, one significant locus with a peak Δ SNP-index value for the G7 dwarf phenotype on chromosome C05 (28.0–28.6 Mb) was identified by MutMap (Fig. 4b). A new dwarf mutant *Bndwf/dcl1* from EMS-mutagenesis has been recently reported. Mutant *Bndwf/dcl1* displayed a sharply down-curved and crinkled phenotype with short petioles at the seedling stage [15]. In the present study, we also observed the same phenotype in the G7 mutant. *Bndwf/dcl1* has been mapped to a 6.58-cM interval on *Bna*C05. Further mapping narrowed the interval of *Bndwf/dcl1* to 175 kb (C05: 29.76–29.94 Mb) in length. This region is adjacent to the peak of the ΔSNP-index value in our study. Putative gene function prediction revealed that one of candidate genes, *BnaC05g29300D* encoding IAA7, has been reported to cause dwarfing and other plant architecture variation in Arabidopsis. Meanwhile, fine mapping of two EMS dwarf mutant also identified causal variation within *Bna.IAA7.C05* [42, 43]. Sequence analysis revealed that these two mutants both have a SNP variation causing an amino acid change in the conserved GWPPV motif (GWPPV to GWLPV) of domain II in *Bna.IAA7.C05*. In present study, the nucleotide variation in conserved motif leads to change of GWPPV to EWPPV. One another EMS mutant of *IAA7* gene in *B. napus* A3 genome also has one SNP in the conserved GWPPV domain (GWPPV to EWLPV) [54]. Thus, this is an allelic mutation in the conserved GWPPV motif of *IAA7* genes in *B. napus* that caused dwarfism phenotype.

In Arabidopsis, the AUX/IAA family contains four conserved domains and exhibit strong gene redundancy, with even triple loss-of-function mutants showing no defects in plant phenotype [26]. Conversely, AUX/IAA gain-of-functional mutations reveal a dramatic change to development as compared to WT. Many gain-of-function mutants of Aux/IAA have been reported in Arabidopsis [39], including those caused by single amino acid substitutions in the conserved GWPPV motif of domain II [55–57]. Gain-of-function mutation of *IAA* mutants in Arabidopsis exhibit decreased apical dominance with shorter stems. The dCAPs marker developed from the sequence variation in the GWPPV motif co-segregated with the dwarf phenotype of G7. Thus, we hypothesize that the dwarf phenotype with down-curved leaf of G7 is most likely to be caused by the G to E amino acid substitution in the GWPPV motif of *Bna.IAA7.C05*.

In wild type plants, auxin will combine with TIR (auxin-transport inhibitor response) when auxin concentration is increased, causing Aux/IAA ubiquitination and degradation. This releases ARFs and eventually their expression is promoted [24]. However, the amino acid change in the GWPPV motif reduces the TIR binding activity and the degradation of Aux/IAA is disrupted, resulting in Aux/IAA protein accumulation and increased repression of ARFs [40]. Small auxin up RNAs (SAUR), are the largest gene family triggered by auxin [58]. SAUR proteins are involved in cell expansion, growth and development of plants [59–61]. In present study, auxin-induced response genes, including many SAURs, were suppressed in the dwarf mutant (Fig. 7b). The low expression level of ARFs and other auxin response genes eventually leads to auxin overproduction and developmental defects.

## Conclusions

In summary, a gain-of-function mutant with dwarfism and down-curved leaf was isolated from tissue culture processes. The candidate region for dwarfism was mapped to a 0.6 Mb region of *B. napus* chromosome C05 through the MutMap method. These results are consistent with previous dwarf mutant mapping results reported in *B. napus*. Further candidate genes analysis revealed that one amino acid substitution from G to E in in the conserved motif GWPPV of *Bna.IAA7.C05* might also lead to dwarfism phenotype. This mutation of *Bna.IAA7.C05* resulted in decreased expression of other *IAA* and auxin response genes expression. Our findings identified one new allele of *Bna.IAA7.C05* responsible for plant dwarf phenotype and provide insights for understanding a dominant dwarfism mutant in *B. napus*.

## Methods

### Plant materials and phenotyping for plant height

The *B. napus* dwarf mutant was originally isolated from tissue culture (Gansu Academy of Agricultural Sciences). A cross was made between 48,557 (wild type, female parent) and the G7 dwarf (pollen donor) to create F_1_ plants which were subsequently selfed to create a segregating F_2_ population. All plant materials were grown at the field in Oil Crops Research Institute of the Chinese Academy of Agricultural Sciences (OCRI-CAAS), Wuhan, China. Plant height was measured when plants attained maximum height at the final flowering stage. DNA samples from 50 of 277 F_2_ plants with the dwarf phenotype were mixed to form the dwarf bulk, and from 50 tall plants to form the tall bulk for MutMap analysis as described by [52].

### Microscopy analysis

Stem segment of *Brassica napus* at the early flowering time stage was fixed by 50% FAA (Formalin–acetic acid–alcohol) solution. Samples were then embedded in Paraffin Plus after dehydration and infiltration steps. Tissues were sliced into about 8 to 10 μm (Leica RM2265) and stained by 0.05% toluidine blue. Image was observed under microscope (Nikon).

### Generation and analysis of NGS data of MutMap analysis

DNA was extracted by DNA sample preparation Kits (Tiangen, Beijing, China) according to the manufacturer’s instructions. The quantification and quality verification was detected by Nanodrop one (Massachusetts, America). About 2 μg genomic DNA from two DNA bulks or two parents were prepared for sequencing library construction. Sequence data were generated by Illumina HiSeq X ten (San Diego, California, USA) with paired-end (PE150) and 350 bp of reads length. Sequence quality and adaptor trimming was conducted by SOAPnuke 1.4 (BGI, Shenzhen, China) and analyzed by Benagen company (Wuhan, China) for both DNA bulks and parental lines. Raw reads including adapter sequences and low-quality reads were removed after processed by Trimmomatic software [45]. Then clean reads with high quality were then aligned to the *B. napus* reference genome by the BWA (Burrows-Wheeler Aligner) software [62–64]. BAM files were created from alignment file by using SAMtools software [65].

### MutMap analysis to determine candidate gene variation

SNP calling and SNP index was performed as previous study reported [52]. After aligned GFF3 files to the *B. napus* genome with ANNOVAR, homozygous SNPs between the dwarf mutant and the normal line were realized from VCF files generated by Variant Filtration-GATK software using defaulting settings [45, 66]. SNP index is the ratio of reads with SNP (harboring nucleotide different to reference genome) to the total reads contained the SNP [52]. The Δ SNP-index was obtained by subtracting the SNP-index between dwarf and normal DNA pools. Sliding window method was conducted to detect the SNP-index across the *B. napus* genome [45]. Δ SNP-index across the chromosome of *B. napus* genome was performed by sliding-window analysis with 1 Mb window size and 10 kb step size [45]. Statistical confidence intervals of the ΔSNP index were defined at 95 and 99% following the description reported before [53, 67].

### Expression analysis of dwarfing candidate genes by semi-quantitative RT-PCR and RT-qPCR

To detect expression pattern of candidate gene, different samples including stem, leaf and floral bud, were taken from five individuals from dwarf mutant G7 and Westar. RNA was extracted by RNAprep Pure kit (Tiangen, China). After quality detection, reverse transcription was conducted by using FastQuant RT kit according to the instruction (Tiangen, China). Semi-quantitative RT-PCR for gene *Bna.IAA7.C05* (*BnaC05g29300D*) was performed for 32 cycles by using the primers (AHC5F and AHC5R) listed in Additional file 2: Table S2. *BnaActin* gene (*BnaC02g00690D*) from *B.napus* was used as the control for the RNA sample. The reaction of semi-quantitative RT-PCR was performed for 32 cycles, with 30 s at 95 °C, 45 s at 57 °C and 50 s at 72 °C. Stem-loop RT-qPCR was used to examine miR169d expression level according to previous method [68]. Stem-loop qRT-PCR was performed in CFX96 Real Time System (Bio-Rad, Hercules, California, USA) using SYBR Green mix (Transgen Biotech, Beijing). The reactions were performed as following program: 30 s at 95 °C, 40 cycles of 5 s at 95 °C, and 30 s at 60 °C. Primers used for stem-loop RT-qPCR were listed in Additional file 2: Table S2.

### RNA-seq of differential gene expression

Seedlings of G7 dwarf and WT (Westar) were selected to extract the RNA. Samples were collected and frozen in liquid nitrogen and stored at − 70 °C for RNA preparation. Total RNA from bulked samples was extracted in accordance with the manufacturer protocol (RNA-kit Tiangen, China). The integrity of the total RNA was detected by 1% agarose gel electrophoresis. All RNA samples were detected by Nanodrop 2000 to analyze A260/A280 value for protein contamination and A230/A280 value for reagent contamination. The concentration was detected by Nano-Drop (Thermo Scientific, La Jolla, CA, USA) and purity of RNA was also checked by Agilent 2100 Bio-analyzer (Agilent-USA). 10 μl RNA sample was used to construct the sequencing library according to manual instruction. Sequence data was generated by Illumina HiSeq X ten (San Diego, California, USA) with paired-end (PE150) and 350 bp of reads length. Sequence quality and adaptor trimming was conducted by SOAPnuke 1.4 (BGI, Shenzhen, China). Clean reads were mapped to the reference genome of *B. napus* by BWA software [62–64].

Transcriptome analysis was mostly conducted as the description in the previous study [68]. False discovery rate (FDR) cutoff of less than 0.01 was used to determine the DEGs. The absolute value of log2 Ratio ≥ 2 between different samples with FDR ≤ 0.001 were determined as DEGs by using DEseq2 software [67]. All annotated genes were mapped to the GO and KEGG database. DEGs in the KEGG pathway were enriched by KOBAS software [69]. GO annotation was performed by Blast2GO software [70]. For each sample, three biological replicates were conducted. The value of log2 transformed FPKM value was used to create heat maps. Color key from yellow to purple represented the expression level with log2 transformed FPKM values. Negative and positive values represented the low and high level, respectively. For each gene, the log2 transformed FPKM value was normalized in each row.

### Statistical analysis

The analysis of RT-qPCR results was carried out by Student’s t-test (*P* < 0.05) or (*P* < 0.01). The RT-qPCR was performed for three biological repeats.

## Supplementary information

**Additional file 1: Table S1.** Genes on the candidate region of chromosome C05 of *Brassica napus.*

**Additional file 2: Table S2.** Primers used in this study.

## Data Availability

All data in this study are included in this published article and its supplementary information files Additional file 1: Table S1 and Additional file 2: Table S2. Sequences data of RNA-seq were deposited to the NCBI Sequence Read Archive (SRA) repository under accession number (SRR10090703, SRR10090704, SRR10090705 for Westar sample; SRR10090706, SRR10090707, SRR10090708 for G7 mutant sample). Genome re-sequencing data could be achieved under accession number (SRR10189121, SRR10189122 for 48557 sample; SRR10189123, SRR10189124 for G7 sample).

## Abbreviations

DEGs: differentially expressed genes
iaa: indole-3-acetic-acid
Mutmap: mutation mapping
SAUR: small up regulated response
